# The role of computational models in mechanobiology of growing bone

**DOI:** 10.3389/fbioe.2022.973788

**Published:** 2022-11-18

**Authors:** Ester Comellas, Sandra J. Shefelbine

**Affiliations:** ^1^ Serra Húnter Fellow, Department of Physics, Universitat Politècnica de Catalunya (UPC), Barcelona, Spain; ^2^ Department of Mechanical and Industrial Engineering and Department of Bioengineering, Northeastern University, Boston, MA, United States

**Keywords:** mechanobiology, endochondral ossification, finite element model (FE model), biomechanics, bone growth and development

## Abstract

Endochondral ossification, the process by which long bones grow in length, is regulated by mechanical forces. Computational models, specifically finite element models, have been used for decades to understand the role of mechanical loading on endochondral ossification. This perspective outlines the stages of model development in which models are used to: 1) explore phenomena, 2) explain pathologies, 3) predict clinical outcomes, and 4) design therapies. As the models progress through the stages, they increase in specificity and biofidelity. We give specific examples of models of endochondral ossification and expect models of other mechanobiological systems to follow similar development stages.

## Perspective

Models of all kinds are used to help understand the world around us. Computer models specifically allow us to digitally reproduce structures or systems that we can use to probe influences and predict consequences. Computational mechanics models, such as finite element modeling, allow a better understanding of the mechanical environment. Biomechanical models examine the mechanical behavior and properties of biological tissues or organs, whereas mechanobiological models explore the effects of the mechanical environment on biological processes and living systems. In both biomechanics and mechanobiological finite element models, the development of the model, often over decades, progresses from a conceptual model, which is used to explore effects and develop hypotheses, to a more specific, realistic model, which is used to predict outcomes. In biomechanics models, this progression may result in increasing complexity of the material properties, more specific loading and boundary conditions, more exact geometry, or more accurate representation of the consequences, such as fracture. In mechanobiological models, simplified conceptual models are used to explore the link between mechanical environment and biological response (e.g. which mechanical stimulus maps spatially and/or temporally to the biological response?). As these models become more complex, they can be used to predict clinical outcomes. The main difference between biomechanics and mechanobiological models is the latter incorporates the biological response by simulating, for example, changes in properties, geometry, or molecular concentrations.

The biological response to mechanics typically results in an altered tissue state and can be modeled at the tissue level as a change in geometry (growth), material properties (matrix production or degradation), or chemical release (diffusion or advection). These biological processes, chemical release, matrix production, and change in structure, are all linked together in complex biological pathways but are often modeled separately in tissue level mechanobiological models. In many, if not most, mechanobiological systems the mechanical stimuli that triggers the response is unknown. At the cellular level, we know that cells can respond to a variety of mechanical stimuli such as stretch, volume change, and fluid flow, and may indeed have a differentiated response to multiple stimuli (Wang and Thampatty, 2006). However, abstracting the effects of mechanics on a single cell or group of cells *in vitro*, to cells in a tissue *in vivo* is challenging, which is precisely the role of computational modeling.

Endochondral ossification is the process by which long bones growth in length. It starts with a cartilage anlage, which becomes vascularized at the primary ossification center near the middle of the bone. The ossification front progresses towards both ends of the bone. At the ossification front, cartilage cells line up in characteristic columns, hypertrophy (grow particularly in the longitudinal direction) and the matrix ossifies to become bone. When the ossification front reaches the end of the shaft, a small nodule of bone forms in the cartilaginous epiphysis, called the secondary center of ossification. The secondary center expands leaving a layer of cartilage, the growth plate, where subsequent growth occurs. Endochondral ossification is influenced by mechanical loading. The biological processes that are influenced by mechanical loading result in a change in shape and material properties, reflecting the growth and ossification of the matrix. Intramembranous ossification is the process by which bones grow in girth with new bone forming directly on bone. Intermembranous ossification is also affected by mechanical loading, but is not discussed in this perspective as it is a separate mechanobiological process.

**FIGURE 1:**
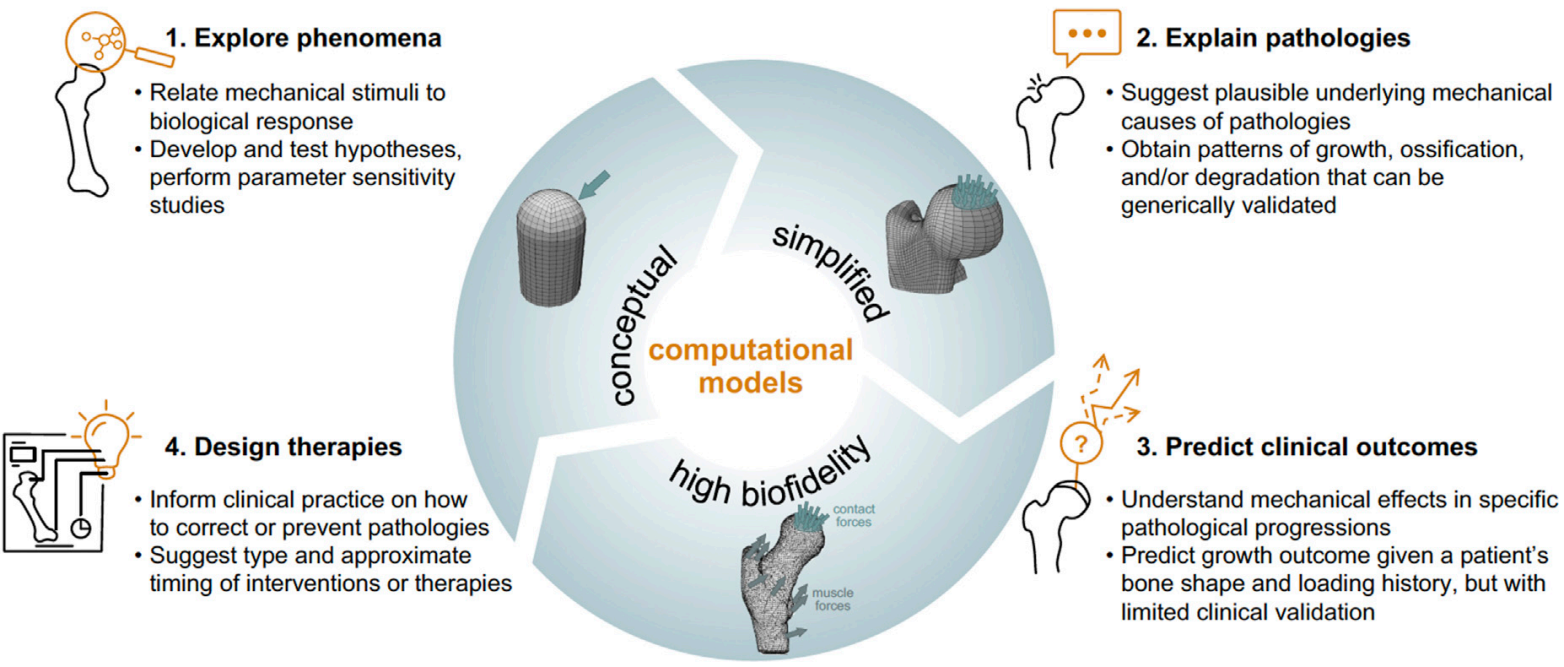
Jpeg.

Mechanobiological models of endochondral growth and ossification have been developed over decades to explore the role of mechanics in regulating this process at the continuum (tissue) level. In this perspective we review the four stages of development of mechanobiological finite element models of endochondral ossification at the tissue level in which models are used to: 1) explore phenomena, 2) explain pathologies, 3) predict clinical outcomes, and 4) design therapies (Figure 1). We discuss uses of the models in each stage and their applications.

Mechanobiological models tend to have a history from conceptual and simplified to specific with high biofidelity. As the models become more specific, they can be used to predict pathologies, simulate patient-specific outcomes, or even to design new therapies. Though this perspective is focused on computational mechanobiological models of endochondral ossification at the tissue level, we believe the life cycle is similar in models of other systems and other length scales.

## Explore phenomena

The first stage of a mechanobiological model is conceptual, consisting of an exploration of possible mechanical stimuli (such as stress, strain, fluid flow) and resulting biological responses (typically change in material properties or change in shape/structure). These models tend to be generic in both geometry and loading conditions, such as a “diarthrodial joint”. Stimuli are explored for patterns of high/low instead of specific magnitudes, thereby making loading directions more critical than applied load magnitudes. In the first models exploring the effects of mechanics on endochondral ossification, mechanical stimuli were explored that could predict formation of the secondary center, a nodule of bone at the end of the joint, and maintenance of cartilage at the joint surface. Finite element models of stresses in the chondroepiphysis found ossification occurred in regions of deviatoric (shear) stress. Cartilage at the joint surface, which does not ossify, experienced hydrostatic compressive stress (Carter and Wong, 1988a). These models highlighted the importance of boundary conditions (earlier photoelastic models of Pauwels (Pauwels, 1960) had opposite conclusions because of faulty boundary conditions). The observation of the relationship between stress state and ossification location led to the formulation of the mechanobiological basis for endochondral ossification, the osteogenic index (*OI*):

$$OI=\sigma_{s}+k\sigma_{h}$$

where σ_s_ is the octahedral shear stress (always positive), σ_h_ is the hydrostatic stress (negative when compressive), and *k* is a proportionality constant. The osteogenic index is a measure of how likely the cartilage will turn to bone. With hydrostatic compressive stress, OI will be low and ossification will be inhibited. With octahedral shear stress, OI will be high and growth and ossification will be promoted. Subsequent studies investigated the influence of *k* (Wong and Carter, 1988; Wong and Carter, 1990; Stevens et al., 1999), loading conditions (Carter and Wong, 1988b) and initial geometries (Wong and Carter, 1988; Giorgi et al., 2014) on the predictions, and developed iterative modeling schemes to update material properties to reflect predicted ossification patterns (Wong and Carter, 1990).

Exploratory models develop hypotheses, test assumptions, study sensitivity to parameters (loading and boundary conditions, material properties, geometry), and develop algorithms and methods for simulating the effects of biological processes within the modeling context (e.g. changing material properties). The models are typically generic and simple, which allows for conceptual hypotheses to be developed and generalized more readily. Exploratory models determine sensitivity to specific parameters, such as *k* in the osteogenic index or cartilage thickness, joint shape, or loading directions. Parameter studies at this stage are critical so that the sensitive parts of the model can be well justified. In this phase of model development there is often not validation. Instead, exploratory models are used to generate mechanobiological hypotheses, which are tested and validated later.

## Explain pathologies

Once mechanobiological theories have been developed from exploratory models, they can be used to suggest the underlying mechanical cause of pathologies. In the musculoskeletal system, altered mechanics results in bone deformities (change in structure) or tissue degradation (change in material properties). Typically, in models used to explain pathology, the model geometry more closely resembles a specific bone of study. Loading conditions are also more specific in order to investigate the influence of altered loading conditions, particularly direction of the loads. Magnitude of the load may play an important role in the amount of bone formation/degradation, but patterns of mechanical stimuli, regions of high or low stimuliz, are more affected by loading directions.

Numerous models have examined the role of mechanical loads in specific pathologies such as explaining the role of loading conditions on the formation of increased neck-shaft angle in developmental dysplasia of the hip (Shefelbine and Carter, 2004a; Giorgi et al., 2015), increased femoral anteversion in children with cerebral palsy (Shefelbine and Carter, 2004b), and articular cartilage thickness and location of osteoarthritis (Beaupré et al., 2000). Models that are used to explain pathology typically compare simulated outcomes between two or more different loading conditions. Because the initial geometry and material properties are similar across models, the effects of loading can be isolated. Models at this stage of development can be generically validated in terms of patterns of growth and ossification, locations of degradation or degeneration, and regions of changing material properties, but are typically not able to predict amount of deformity or degeneration nor the speed with which pathology progresses. Phenomenological models that assume tissue level stimuli result in tissue level responses, ignore the underlying biochemical transduction of the mechanical stimuli and assume “all else is equal”. In reality, loading conditions cannot be isolated from other factors that may affect the biological response such as nutrition, blood supply, and hormones. Nonetheless, predictive models of pathology can provide suggestions as to plausible mechanical causes of pathology, which can help to identify underlying cellular and molecular mechanisms.

## Predict clinical outcomes

Once the simplified models have suggested potential mechanical factors that affect skeletal growth and ossification, the model can be adapted to validate the previously formed pathological hypotheses through prediction of clinical outcomes, requiring an increase in accuracy and specificity of the model. For these models, geometry can be obtained from CT or MR images. Approximate loading conditions are insufficient and specific loading conditions from musculoskeletal models are typically used. Instead of a single loading condition, a range of loading conditions is used to better represent a loading history. Increasing the fidelity of the model allows for specific patient modeling, and asking “if this patient were to move like this, how would the bone respond?”

Modeling has been used to predict shape changes in the proximal femur during normal growth, using MR images to inform bone and growth plate shape of the finite element model and gait analysis combined with musculoskeletal modeling to inform the loading conditions (Yadav et al., 2016). Models have been adapted to understand effects of specific gait abnormalities on bones of children with cerebral palsy (CP), who suffer numerous bone deformities (Carriero et al., 2011; Kainz et al., 2020). In order to validate the predicted clinical outcomes, it is required to have longitudinal, patient-specific studies. Significant changes in bone morphology happen over years, during which time patients are often treated, limiting the ability to validate. However, such models may be helpful in explaining why certain children suffer progressive deformity with treatment while others do not. Kainz et al. predicted growth of the proximal femur from stresses due to gait in children with CP and typically developing children (Kainz et al., 2021). Some children with CP had predicted growth patterns similar to typically developing children while other children with CP had predicted growth patterns indicating progressive deformities. The study found sagittal gait pathologies, such as knee and hip flexion, were critical to predicting abnormal growth, which provides a potential avenue for therapy.

As the models become more patient-specific, both the geometry and loading conditions become critical. The first musculoskeletal models examining bone growth in children with cerebral palsy used a scaled adult femur geometry for both musculoskeletal and finite element models (Carriero et al., 2011). The next models used a scaled adult for the musculoskeletal model and an accurate child geometry for the finite element model (Yadav et al., 2016). Then, a pipeline was developed to use accurate geometry from MR images for both the musculoskeletal and finite element model (Kainz et al., 2020). The geometry of bones in the musculoskeletal model was critical to the predicted joint loads and muscle forces (Scheys et al., 2008a; Scheys et al., 2008b; Lenaerts et al., 2009; Scheys et al., 2011). This indicates that specificity is often required in all aspects of the model, particularly when predicting specific clinical outcomes.

## Design therapies

If models can tell us why a pathology happens and can predict progression of the pathology, can models be used to develop therapies to correct or prevent the pathology? Models of bone growth are not quite to this point yet. However, the future will be to use the models to develop new therapies, braces, or exercise regimes to prevent bone deformities in growing bone. For example, we know normal walking results in a decrease in femoral anteversion angle during typical growth (Beals, 1969; Fabry et al., 1973). Children with cerebral palsy have increased anteversion (Laplaza et al., 1993) likely caused by altered gait (Shefelbine and Carter, 2004b; Carriero et al., 2011). Can we use models to determine the critical loads required for decreased anteversion? Once we understand the critical loads, we can develop exercise regimes that deliver those loads to the developing femur. Models will not tell us how much exercise is required, but could inform which exercises are likely to be most effective. Models could also help in determining timing of therapy. For example, models recently showed that wearing a Pavilk harness an additional 6 weeks did not improve outcomes for infants with dysplasia of the hip (Sadeghian, 2022). Models may also help us to understand when therapy may be most critical. For example, a bump (cam) appears on the anteriosuperior aspect of the proximal femur in elite adolescent athletes of particular sports during bone growth (Morris et al., 2018). This condition results in femoroacetabular impingent syndrome and eventual hip osteoarthritis. Models could be used to understand timing of interventions to prevent cam formation.

Is there a place for models directly in clinical practice? Likely not, as the information from models may not be directly useful to the clinic. However, models could certainly inform clinical practice, which is the role of research informing clinical guidelines. As we start to use models to develop clinical therapies, the models will likely enter the model lifecycle again: simplified to understand relationships, exploratory to examine potential therapeutic options, and specific to predict the clinical effects of therapy. Similar to the iterative design process, models can and should be iterative. Increasing complexity is not always necessary to advance a model.

## Summary

Models can also help us explore how things work. As a model develops, it increases in complexity, biofidelity, and specificity, which makes it more useful for clinical application. However, the generic, conceptual, simplified mechanobiological models are critical for understanding the basic relations between mechanics and biological processes. Simple models can help to identify sensitive parameters, explore potential stimuli, and investigate the effects of boundary and loading conditions. This knowledge can then be carried through to the more complex, personalized, biofidelic models. We have outlined here how models of endochondral ossification have progressed through the model lifecycle, but there are many other examples of mechanobiological models that follow similar model development paths: fracture healing, bone modeling/remodeling, cardiovascular flow, and muscle growth. We hope this perspective helps in highlighting the importance of every stage of model development.

## Data Availability

The original contributions presented in the study are included in the article/Supplementary Material, further inquiries can be directed to the corresponding author.

## References

[B1] Beals R. K. (1969). Developmental changes in the femur and acetabulum in spastic paraplegia and diplegia. Dev. Med. Child. Neurol. 11, 303–313. 10.1111/j.1469-8749.1969.tb01437.x 5794162

[B2] Beaupré G. S. Stevens S. S. Carter D. R. (2000). Mechanobiology in the development, maintenance, and degeneration of articular cartilage. J. Rehabil. Res. Dev. 37, 145–151. 10850820

[B3] Carriero A. Jonkers I. Shefelbine S. J. (2011). Mechanobiological prediction of proximal femoral deformities in children with cerebral palsy. Comput. Methods Biomech. Biomed. Engin. 14, 253–262. 10.1080/10255841003682505 20229379

[B4] Carter D. R. Wong M. (1988). Mechanical stresses and endochondral ossification in the chondroepiphysis. J. Orthop. Res. 6, 148–154. 10.1002/jor.1100060120 3334736

[B5] Carter D. R. Wong M. (1988). The role of mechanical loading histories in the development of diarthrodial joints. J. Orthop. Res. 6, 804–816. 10.1002/jor.1100060604 3171761

[B6] Fabry G. MacEwen G. D. Shands A. R. (1973). Torsion of the femur. J. Bone Jt. Surg. 55, 1726–1738. 10.2106/00004623-197355080-00017 4804993

[B7] Giorgi M. Carriero A. Shefelbine S. J. Nowlan N. C. (2015). Effects of normal and abnormal loading conditions on morphogenesis of the prenatal hip joint: Application to hip dysplasia. J. Biomech. 48, 3390–3397. 10.1016/j.jbiomech.2015.06.002 26163754 PMC4601017

[B8] Giorgi M. Carriero A. Shefelbine S. J. Nowlan N. C. (2014). Mechanobiological simulations of prenatal joint morphogenesis. J. Biomech. 47, 989–995. 10.1016/j.jbiomech.2014.01.002 24529755

[B9] Kainz H. Killen B. A. Van Campenhout A. Desloovere K. Garcia Aznar J. M. Shefelbine S. (2021). ESB Clinical Biomechanics Award 2020: Pelvis and hip movement strategies discriminate typical and pathological femoral growth - insights gained from a multi-scale mechanobiological modelling framework. Clin. Biomech. (Bristol, Avon. 87, 105405. 10.1016/j.clinbiomech.2021.105405 34161909

[B10] Kainz H. Killen B. A. Wesseling M. Perez-Boerema F. Pitto L. Garcia Aznar J. M. (2020). A multi-scale modelling framework combining musculoskeletal rigid-body simulations with adaptive finite element analyses, to evaluate the impact of femoral geometry on hip joint contact forces and femoral bone growth. PloS One 15, e0235966. 10.1371/journal.pone.0235966 32702015 PMC7377390

[B11] Laplaza F. J. Root L. Tassanawipas A. Glasser D. B. (1993). Femoral torsion and neck-shaft angles in cerebral palsy. J. Pediatr. Orthop. 13, 192–199. 8459010

[B12] Lenaerts G. Bartels W. Gelaude F. Mulier M. Spaepen A. Van der Perre G. (2009). Subject-specific hip geometry and hip joint centre location affects calculated contact forces at the hip during gait. J. Biomech. 42, 1246–1251. 10.1016/j.jbiomech.2009.03.037 19464012

[B13] Morris W. Z. Li R. T. Liu R. W. Salata M. J. Voos J. E. (2018). Origin of cam morphology in femoroacetabular impingement. Am. J. Sports Med. 46, 478–486. 10.1177/0363546517697689 28334547

[B14] Pauwels F. (1960). A new theory on the influence of mechanical stimuli on the differentiation of supporting tissue. The tenth contribution to the functional anatomy and causal morphology of the supporting structure. Z. Anat. Entwicklungsgesch. 121, 478–515. 14431062

[B15] Sadeghian M. (2022). A computational model of endochondral ossification in long bones. PhD Thesis. Boston (MA): Northeastern University.

[B16] Scheys L. Desloovere K. Suetens P. Jonkers I. (2011). Level of subject-specific detail in musculoskeletal models affects hip moment arm length calculation during gait in pediatric subjects with increased femoral anteversion. J. Biomech. 44, 1346–1353. 10.1016/j.jbiomech.2011.01.001 21295307

[B17] Scheys L. Spaepen A. Suetens P. Jonkers I. (2008). Calculated moment-arm and muscle-tendon lengths during gait differ substantially using MR based versus rescaled generic lower-limb musculoskeletal models. Gait Posture 28, 640–648. 10.1016/j.gaitpost.2008.04.010 18534855

[B18] Scheys L. Van Campenhout A. Spaepen A. Suetens P. Jonkers I. (2008). Personalized MR-based musculoskeletal models compared to rescaled generic models in the presence of increased femoral anteversion: Effect on hip moment arm lengths. Gait Posture 28, 358–365. 10.1016/j.gaitpost.2008.05.002 18571416

[B19] Shefelbine S. J. Carter D. R. (2004). Mechanobiological predictions of femoral anteversion in cerebral palsy. Ann. Biomed. Eng. 32, 297–305. 10.1023/b:abme.0000012750.73170.ba 15008378

[B20] Shefelbine S. J. Carter D. R. (2004). Mechanobiological predictions of growth front morphology in developmental hip dysplasia. J. Orthop. Res. 22, 346–352. 10.1016/j.orthres.2003.08.004 15013095

[B21] Stevens S. S. Beaupré G. S. Carter D. R. (1999). Computer model of endochondral growth and ossification in long bones: Biological and mechanobiological influences. J. Orthop. Res. 17, 646–653. 10.1002/jor.1100170505 10569472

[B22] Wang J. H-C. Thampatty B. P. (2006). An introductory review of cell mechanobiology. Biomech. Model. Mechanobiol. 5, 1–16. 10.1007/s10237-005-0012-z 16489478

[B23] Wong M. Carter D. R. (1990). A theoretical model of endochondral ossification and bone architectural construction in long bone ontogeny. Anat. Embryol. 181, 523–532. 10.1007/BF00174625 2396753

[B24] Wong M. Carter D. R. (1988). Mechanical stress and morphogenetic endochondral ossification of the sternum. J. Bone Jt. Surg. 70, 992–1000. 10.2106/00004623-198870070-00006 3403589

[B25] Yadav P. Shefelbine S. J. Gutierrez-Farewik E. M. (2016). Effect of growth plate geometry and growth direction on prediction of proximal femoral morphology. J. Biomech. 49, 1613–1619. 10.1016/j.jbiomech.2016.03.039 27063249

